# Smart healthcare IoT applications based on fog computing: architecture, applications and challenges

**DOI:** 10.1007/s40747-021-00582-9

**Published:** 2021-11-17

**Authors:** Vu Khanh Quy, Nguyen Van Hau, Dang Van Anh, Le Anh Ngoc

**Affiliations:** 1 Hung Yen University of Technology and Education, Khoai Chau, Hungyen Vietnam; 2 Swinburne Vietnam, FPT University, Hanoi, Vietnam

**Keywords:** Fog computing, Cloud computing, Internet of Things, Healthcare

## Abstract

The history of human development has proven that medical and healthcare applications for humanity always are the main driving force behind the development of science and technology. The advent of Cloud technology for the first time allows providing systems infrastructure as a service, platform as a service and software as a service. Cloud technology has dominated healthcare information systems for decades now. However, one limitation of cloud-based applications is the high service response time. In some emergency scenarios, the control and monitoring of patient status, decision-making with related resources are limited such as hospital, ambulance, doctor, medical conditions in seconds and has a direct impact on the life of patients. To solve these challenges, optimal computing technologies have been proposed such as cloud computing, edge computing, and fog computing technologies. In this article, we make a comparison between computing technologies. Then, we present a common architectural framework based on fog computing for Internet of Health Things (Fog-IoHT) applications. Besides, we also indicate possible applications and challenges in integrating fog computing into IoT Healthcare applications. The analysis results indicated that there is huge potential for IoHT applications based on fog computing. We hope, this study will be an important guide for the future development of fog-based Healthcare IoT applications.

## Introduction

History demonstrated that the evolution of humanity goes hand in hand with the development and application of science and technology in the field of medicine and healthcare. Decades ago, information technology applications in the medical area were invented to collect, monitor and control the patient status remotely, decision-making treatment for patients [1–3]. With the development of science came the advent of cloud computing technology. Cloud technology enables to provide all things such as services, include Infrastructure as a Service (IaaS), Platform as a Service (PaaS), and Software as a Service (SaaS). Robustness, reliability, and powerful computing abilities have made cloud computing the dominant technology of the past decade [4, 5].

**Fig. 1:**
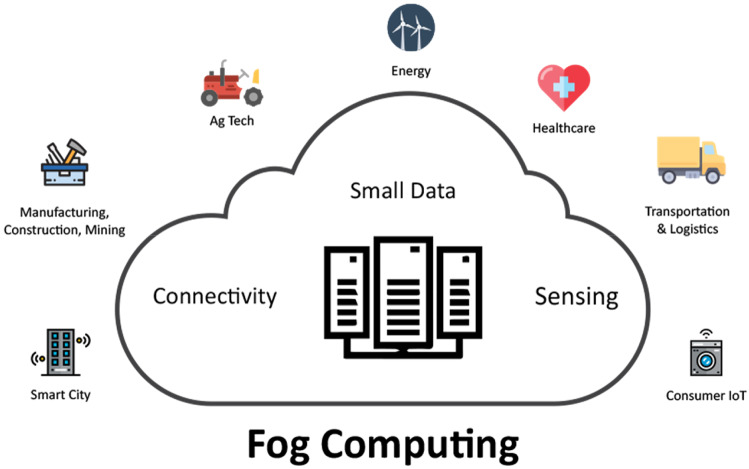
An illustration of fog computing-based applications for humanity

In the early 2020s, the advent of 5th generation mobile networks, also known as 5G, brought a new revolution to all industries. 5G allows providing services with ultra-low latency and ultra-high bandwidth [6, 7]. The advent of 5G realizes the concept of the Internet of Things and forms a series of smart applications serving humanity such as smart healthcare [8], intelligent transportation systems [9], smart cities [10], smart retail [11], smart agriculture [12], and IoT ecosystems [13, 14] as presented in Fig. 1.

In a typical electronic healthcare system (e-Health), sensors are mounted on the body of patients. These devices collect data such as blood pressure, heart rate, mobility, etc. and transmit the collected data to the centre based on network connections [15]. Specifically, in [16], the authors introduced a semantic sensor network to enhance the interoperability ability of sensors in e-health systems for the purpose of the collected different e-Health data formats. Due to the amount of data collected being huge, they will be stored in the cloud for analysis and decision-making [17]. However, one challenge of cloud-based applications is high service response time [18]. In ambulance emergency scenarios, where related data collection such as hospital location, distance, ambulance vehicles, medical staff, etc. The decision-making will be calculated in seconds and directly affected the patient life, cloud computing is not feasible. A computational technology with truly real**-**time response times could save the life of patients. In recent times, fog computing has been proposed [19–21]. Fog computing brings cloud capabilities into LAN to reduce service response time.

In this study, we make a comparison between computing technologies. Then, we present a common architectural framework for fog computing-based IoT applications. Besides, we also point out possible applications and challenges in integrating edge computing into IoT Healthcare applications. Some of the main contributions in this study are described as follows:

- Vision of fog computing and IoT in healthcare industry.
- Compare of cloud, edge, and fog computing.
- A short survey of fog computing-based healthcare IoT applications.
- Applications, challenges and future research directions.

The rest of the paper is organized as follows: in Sect. 2, we present a common fog computing architecture framework. In Sect. 3, we present a short survey of recent fog computing-based Internet of Health applications. Section 4 presents challenges and future research directions, and Sect. 5 is the conclusion.

## Computing architectures

For decades now, cloud computing has dominated all domains [22, 23]. A typical Cloud-based architecture consists of two layers: the Cloud layer consists of cloud servers that have robust storage and high-performance computing ability. They communicate with each other base on the core optical network with extremely bandwidth and the end-user layer consist of end-user devices.

These devices are connected with each other and communicate with the Cloud layer based on wired or wireless connections. Due to the computing, analysis and storage are performed on the Cloud, these devices without need the storage and computing abilities. Cloud computing has the capability of providing all things as a service, including infrastructure as a service, platform as a service, and software as a service [24]. Robustness, reliability and massive computing power make cloud computing to continue the core computing technology of the future [25, 26].

One of the significant limitations of cloud computing is the high service response time. Consequently, it is infeasible for real-time IoT applications, especially for delay-sensitive applications in some areas such as augmented reality [27], traffic [28, 29], and emergency [30]. In recent times, to address these challenges, edge computing [31] and fog computing [32] are proposed.

**Fig. 2:**
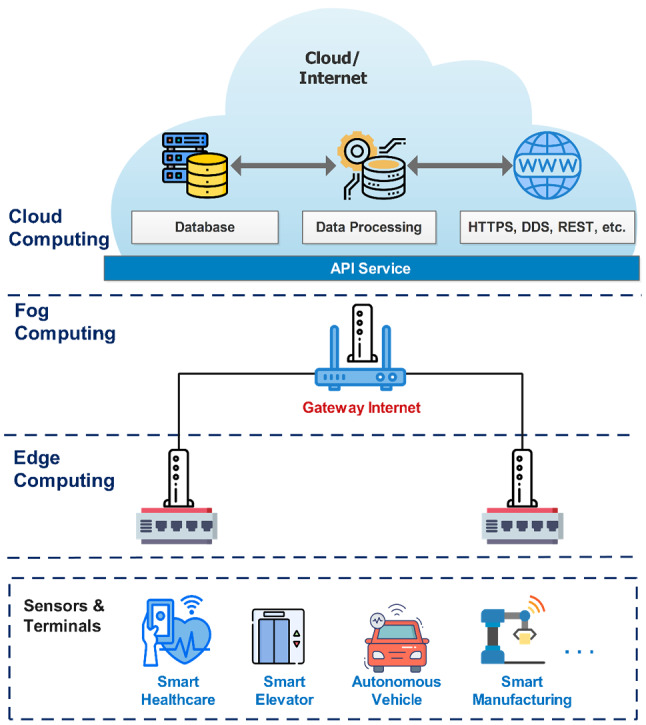
An illustration of all-in-one computing architectures

We present an all-in-one computing architecture as in Fig. 2. These edge and fog technologies both tend the shorter distance between the database and the end-user.

One of the key differences between the two computing technologies is performance.

Edge computing sets up computing servers at the gateway of the network while fog computing brings compute servers into the LAN. On the other hand, fog is the way to bring cloud capabilities to the edge of the network [33, 34]. About nature, fog is the standard, and the edge is the concept. The difference between fog computing and edge computing is only implement performance. In some cases, these two concepts overlap. To explain the capabilities of the computing technologies, in [33] presents a performance comparison of computing technologies as in Table 1.

**Table 1:** Comparison of computing methods

Characteristics	Cloud	Fog	Edge
Latency	High	Low	Low
Service response time	High	Very low	Low
Bandwidth	High	Low	Very low
Storage	High	Low	Low
Server overhead	Very high	Low	Very low
Network congestion	High	Low	Low
Energy consumption	High	Low	Low

In this comparison, we indicate some key criteria that directly affect system performance as follows:

- *Latency:* it is the average time to transmit a packet between two network nodes. Latency criteria reflect the transmission capacity and system performance.
- *Service Response Time:* it is the average time from end-user request service to the data is responded. Service Response Time criteria reflect the accuracy, reliability, and real-time response ability of IoT services.
- *Bandwidth:* this criterion reflects the average bandwidth consumption when processing one request of each solution.
- *Storage:* this criterion reflects the data storage capacity of each solution.
- *Server Overhead:* this criterion reflects the average computational cost per request of each solution.
- *Network Congestion:* this criterion reflects the probability of collision of the packets.
- *Energy Consumption: *this criterion reflects the average energy consumption of each solution.

**Fig. 3:**
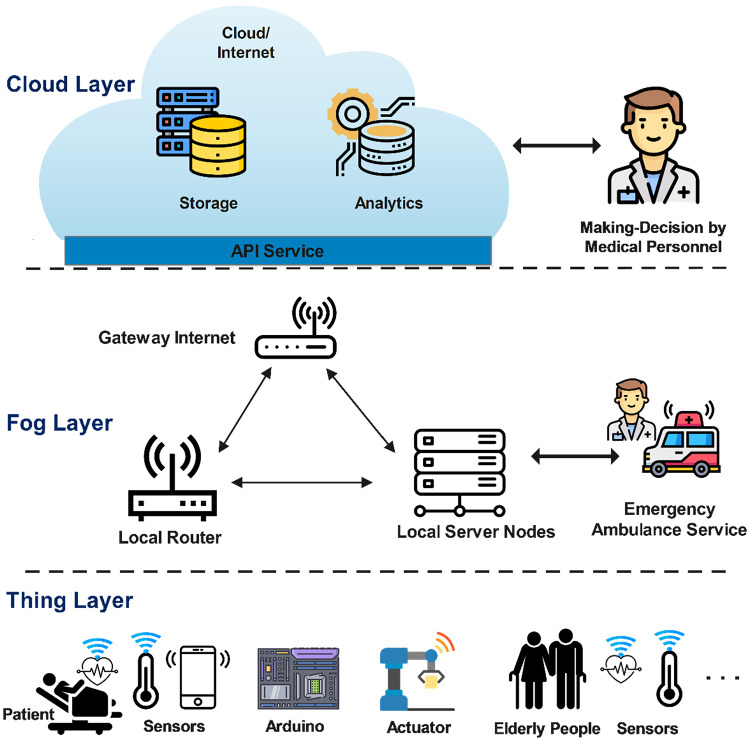
An overview architecture of fog-based IoHT applications

Figure 3 presents an overview architecture of fog-based IoHT applications. This architecture consists of three layers: Thing layer, Fog layer, and Cloud layer. Thing layer includes end-user devices such as sensors, wearable IoT devices, Arduino motherboards, actuators to collect body data, heart rate, etc. The Fog layer includes the Internet gateway, local router, and fog servers. This layer communicates and transfers data between the Thing and Cloud layers. It uses provided data from the underlayer, federated learning, fog computing, and deep learning to making-decisions for emergency medical scenarios. The Cloud layer includes servers with extreme huge storage, processing and analysis capabilities that help medical staff make long-term treatment decisions for patients.

Obviously, with very low service response time, fog computing will be one of the most feasible solutions for Internet of Healthcare Things applications in emergency rescue scenarios and in some real-time patient monitoring applications such as patients with a history of heart disease, blood pressure and stroke. The common feature of these applications is that they require real-time decision-making and response times.

## Survey of recent IoT healthcare applications

The history of human development has proven that medicine and healthcare are the main driving forces for the development of science and technology [35, 36]. Cloud-based medical applications have grown robustly in recent years [37, 38]. The advent of 5G has realized the concept of the Internet of Things and forces the further development of IoHT applications. In recent times, for the purpose to reduce service response time, improve system performance, and energy efficiency, integrating fog computing into the IoHT applications are proposed and achieved some positive results. In this section, we present the fog computing-based proposals in recent three year ranges [2019–2021]. Based on the survey results, we divide the proposals into three categories based on approach, include *Performance*, *Security*, and *Offloading*. Moreover, we also show the method, the purpose and the achieved results with each proposal. The details of the survey results are presented in Table 2.

**Table 2:** The fog-IoHT proposals in period [2019–2021]

Ref	Year	Approach	Purpose	Method	Accuracy	Delay	Energy	Security	Results
[39]	2019	Performance	Fog-IoHT to monitoring and diagnose the hypertension	Use ANN predict the hypertension attack and making-decision based on fog computing	Yes	Yes	No	No	Improve performance, and response time
[40]	2019	Security	Privacy ensured e-healthcare framework to secure patient information records	Use novel consensus-based access control method to ensure the reliability of the requester to EMR	No	Yes	No	Yes	Improve privacy of patient information record, and response time
[41]	2020	Security	Fog-IoHT to enhance efficiency and security	Use user authentication method through identity and elliptic curve cryptography technique to prevent security breaches	No	Yes	No	Yes	Improve performance and enhance security
[42]	2020	Performance	Fog-IoHT to reduced patient data retrieval time	Use the in-network caching of NDN and request aggregation of the content-centric networking	No	Yes	Yes	No	Reduce 28.5% patient data retrieval time
[43]	2020	Performance	Fog-IoHT architecture to saving and optimise energy consumption	Use the MILP method to optimize the number and location of fog devices at the network edge	No	Yes	Yes	No	Saving energy up to [36–52]% compared to Cloud-IoHT systems
[44]	2020	Performance	Fog-IoHT analyzes and identifies heart diseases automatically	Integrates deep learning with fog computing	No	Yes	No	No	Improve the execution time up to [10–43]% under different scenarios
[45]	2021	Offloading	Fog-IoHT offloading schema to optimal offload plan	Use the MSSP method to minimizing the total latency of offloading as well as consider how offloading perform	No	Yes	Yes	No	This solution can rapidly give to the approximate optimal results
[46]	2021	Security	Blockchain-fog-IoHT to improve privacy, security, and diagnostic accuracy diabetic and cardio disease	Use a blockchain to store patient health information and a neuro-fuzzy inference system to making-decision	Yes	Yes	No	Yes	Improve privacy and security; enhance diagnostic accuracy up to 81%
[47]	2021	Offloading	Fog-IoT to enhance performance the diagnosis and treatment of infected patients Coronavirus	Use non-linear and non-convex approaches to solve the suboptimal low-complexity problems	No	No	Yes	No	Reduce computing cost and response time
[48]	2021	Performance	Fog-IoHT to monitoring physical status of athletes	Use the 3D-acceleration method to record the collected health data state of athletes for workout activity	Yes	No	No	No	Improve health of athletes; provide timely warnings; gym activity recognition
[49]	2021	Offloading	Fog-IoHT to adaptive for large-scale healthcare applications	Use load balancing method to offloading among fog servers nodes	No	Yes	No	No	Improve performance compared to the Cloud systems
[50]	2021	Security	Fog-IoHT based on symmetric homomorphic cryptosystem to secure patient information	Use an symmetric homomorphic cryptosystem to privacy assured medical data aggregation	No	Yes	No	Yes	Improve privacy and security
[51]	2021	Performance	Fog-IoHT to reduce the delay, bandwidth and energy, enhance the reliability of system	Use a MOCSA algorithm to solve the multi‑objective optimization problem of both energy and latency	No	Yes	Yes	No	Improve the latency and saving energy up to [5–42]% and [28–43]%, respectively compared to several existing solutions

Hypertension is a very dangerous disease, it often occurs in the elderly or people with a history of the disease. A hypertension attack can suddenly appear. Without the timely support of relatives and doctors, the patient's life will be at risk. To solve this problem, in [39], Sood et al. proposed an IoT healthcare application based on fog computing to monitoring and diagnose a hypertension attack of the patient in real-time. The operating principle of the system is as follows: they use sensors mounted on the patient to collect blood pressure data. Then, an artificial neural network will be used to predict blood pressure attacks based on fog computing. The system will continuously generate blood pressure alerts to pre-installed mobile devices. Experiment results demonstrated the proposed solution reduced service response time, bandwidth consumption, and enhance the accuracy of diagnosis compared to existing solutions.

In recent times, the explosion of healthcare IoT applications based on fog computing presented the huge potential of fog computing. However, on the other hand, these applications make a significant challenge in protecting the privacy and security of patient health information. To solve these challenges, Saha et al. [40] proposed the privacy ensured e-healthcare framework for electronic medical records in healthcare IoT applications based on fog computing. Experimental results demonstrated the efficiency of the proposed solution in terms of the privacy and security of patient information, reduced response time compared to existing solutions.

Realizing the limitations of healthcare IoT applications based on the Cloud, Awaisi et al. [41] proposed an efficient healthcare IoT based on fog computing to enhance efficiency and security. Specifically, they proposed an efficient healthcare IoT architecture based on fog computing. Then, to enhance security, they use the identity-based user authentication method. Experiment results indicated the efficiency of the proposed solution in terms of performance and enhance security.

One of the main challenges facing healthcare applications is the ability and speed of patient data retrieval. The limited capabilities and capacities of IoT nodes are not viable. To address this challenge, Wang et al. [42] proposed a healthcare IoT system based on fog computing to reduce patient data retrieval time. Specifically, they exploit caching and request aggregation of the content centric networking. Experiment results indicated that the proposed solution improved up to 28.5% in terms of the patient data retrieval time compared to existing solutions.

The robust increase of Cloud-based healthcare IoT applications leads to the consumption of amount huge energy. According to energy efficiency direction, Isa et al. [43] proposed a fog computing-based architecture for healthcare IoT applications to saving and optimise energy consumption. Specifically, they proposed an efficient energy fog-based computing model, called EEFC to optimize the location and number of fog servers at the edge layer. Experiment results demonstrated the efficiency of the proposed solution compared to existing cloud-based solutions when it saving energy up to 36% and 52%, respectively with low and high data speed scenarios.

Cloud-based IoT applications have been successfully deployed in the last decade, however, Shreshth Tuli et al. [44] demonstrated that existing IoHT applications based on the cloud have limited scalability and high service response time. To solve these challenges, the authors proposed a novel IoHT architecture based on fog computing and deep learning to real-time analysis and diagnostic for heart patients. Simulation results indicated that the proposed framework improved in terms of energy consumption, bandwidth, accuracy, delay, and execution time in different fog-based IoHT scenarios.

The integration of fog computing into healthcare IoT applications aim to make these real-time eHealth applications is the inevitable trend. However, the breakout of IoT devices will lead to overload. To solve this problem, Zhang et al. [45] proposed an efficient offloading schema for fog servers in real-time eHealth systems.

Specifically, they consider the offloading problem as multi-stage stochastic programming that aims to minimize the total delay to determine the optimal offload plan based on system resources. Experiment results indicated that the proposed solution can rapid be led to the approximate optimal results.

For the purpose design a privacy and security healthcare IoT system to diagnostic accuracy diabetic and cardio disease, Shynu et al. [46] proposed an integrated solution between healthcare IoT application, blockchain and fog computing technologies. Specifically, sensors will collect patient data and storage on the blockchain. Then the patient health records will be grouped, and finally, an adaptive neuro fuzzy inference system is used for making-decision diagnostic based on fog computing. Experiment results indicated that the efficiency of the proposed solution when it improved accurate diagnosis rate up to 81% compared to existing solutions.

In the context of the outbreak of the COVID-19 epidemic, the Internet of Medical Things (IoMT) plays an important role in supporting the diagnosis and treatment of patients infected with the coronavirus. To enhance performance and reduced service response time of these applications. In [47], Qiu et al. proposed an IoMT based on fog computing. Specifically, they use an efficient offloading schema to resource allocation between fog servers aims to limited overload. Experiment results indicated that the proposed solution improved computing cost and reduce response time compared to existing solutions.

According to the development trend of society, more citizens go to gyms to exercise. The monitoring activities, exercises, and body compositions of athletes to give warnings timely or provide analyses of the change of body signals to doctors, coaches, and athletes are urgent requirements. To solve this problem, Hussain et al. (2021) [48] proposed a fog-based healthcare IoT framework. Specifically, they use wearable IoT devices to collect body data, exercise intensity, heart rate. Then, athlete information records are stored, analyzed at fog servers to make optimize exercise, duration, and intensity for each athlete. Through experiment, they constructed three modules, include Health zone to classify the health of athletes, Hzone to provide timely warnings about the health status of athletes, and a gym activity recognition module. Experiment results demonstrated the efficiency of the proposed solution compared to existing solutions.

High service response delay is one of the limitations of Cloud-based healthcare IoT applications. To solve this problem, Asghar et al. [49] proposed a fog-based healthcare IoT architecture framework. Moreover, to adaptive for large-scale healthcare applications, they also introduce an efficient offloading schema to load balancing between fog servers. Experiment results based on iFogSim simulation software indicated that the proposed solution reduced response time compared to the Cloud-based approach.

In the COVID-19 pandemic, fog-based healthcare IoT applications showed their important position and inevitable development trend in the future. However, the analysis of patient information records at the fog servers faces privacy and security challenges. To address these challenges, Guo et al. [50] proposed an improved symmetric homomorphic cryptosystem for monitoring delay-sensitive applications. Finally, they experimented with their solution with two attack games to indicate the improved privacy and security. Experiment results demonstrated the efficiency of the proposed solution compared to existing related approaches.

Although fog computing has great potential to provide network resources and requested services for applications at the edge network. However, inefficient deployment of components on fog infrastructure results bandwidth, resource and energy wastage, reduce system performance. To solve this problem, Yaser Ramzanpoor et al. [51] proposed a multi‑objective optimization algorithm for fog-IoHT applications. Authors used a multi-objective cuckoo search algorithm (MOCSA) to solve the multi‑objective optimization problem of both energy and latency. The simulation results show that the proposed solution improved average latency the amount [5–42]% and reduce energy consumption about [28–43]% compared to several existing solutions.

## Discussion and applications

Analysis of the surveyed results in Table 2 presented some interesting results. The proposals focus on three main approaches, including *performance improvement*, *privacy and security*, and *offloading*.

According to the *performance improvement* approach, fog-IoHT applications focus on reducing service response time, energy efficiency, and improving the accuracy of decision-making. Some typical results are as follows: in [42], authors reduced the patient data retrieval time by 28.5% compared to existing solutions. Moreover, in [43], authors improved energy consumption up to 52% compared to Cloud-based systems in high data speed scenarios.

According to the *privacy and security* approach, fog-IoHT applications focus on ensuring privacy and data security at fog servers. Information related to the health condition of patients is particularly sensitive and needs to be private. Ensuring information security for healthcare-related applications is an important task. Obviously, data storage and processing at fog servers brings unprecedented abilities and capabilities to IoHT applications. On the other hand, it also brings the attacked potential risks on fog servers. Therefore, a series of proposals to improve privacy and security for IoHT have been proposed over the years in [2, 3, 7, 11].

In [52], authors proposed a secure edge computing solution for IoT applications based on the security gateway that is deployed on edge to manage IoT devices, data, IoT clients, and services aim to provide secure edge services of access and visualization to users.

According to *offloading* approach, fog-IoHT applications focus on offloading for fog servers. The explosive growth of sensors, IoT devices and smart healthcare applications generates a huge amount of data. Consequently, it will put pressure and overload on fog computing servers. An effective offloading strategy is an inevitable requirement and one of the most significant challenges for real-time fog-IoHT applications.

It should be recalled that, although *performance improvement* and *efficient offloading* all have the same goals to reduce service response time, energy, and enhance the accuracy of decision-making, they have two different approaches. While *performance improvement* uses different solutions and methods to optimize performance criteria on a fog server, *efficient offloading* focuses on establishing an optimal assignment of tasks schema for fog servers. In [6], the authors achieved an approximate optimal offloading schema. Some other studies in [8, 10] improved computing costs and reduced response times.

**Fig. 4:**
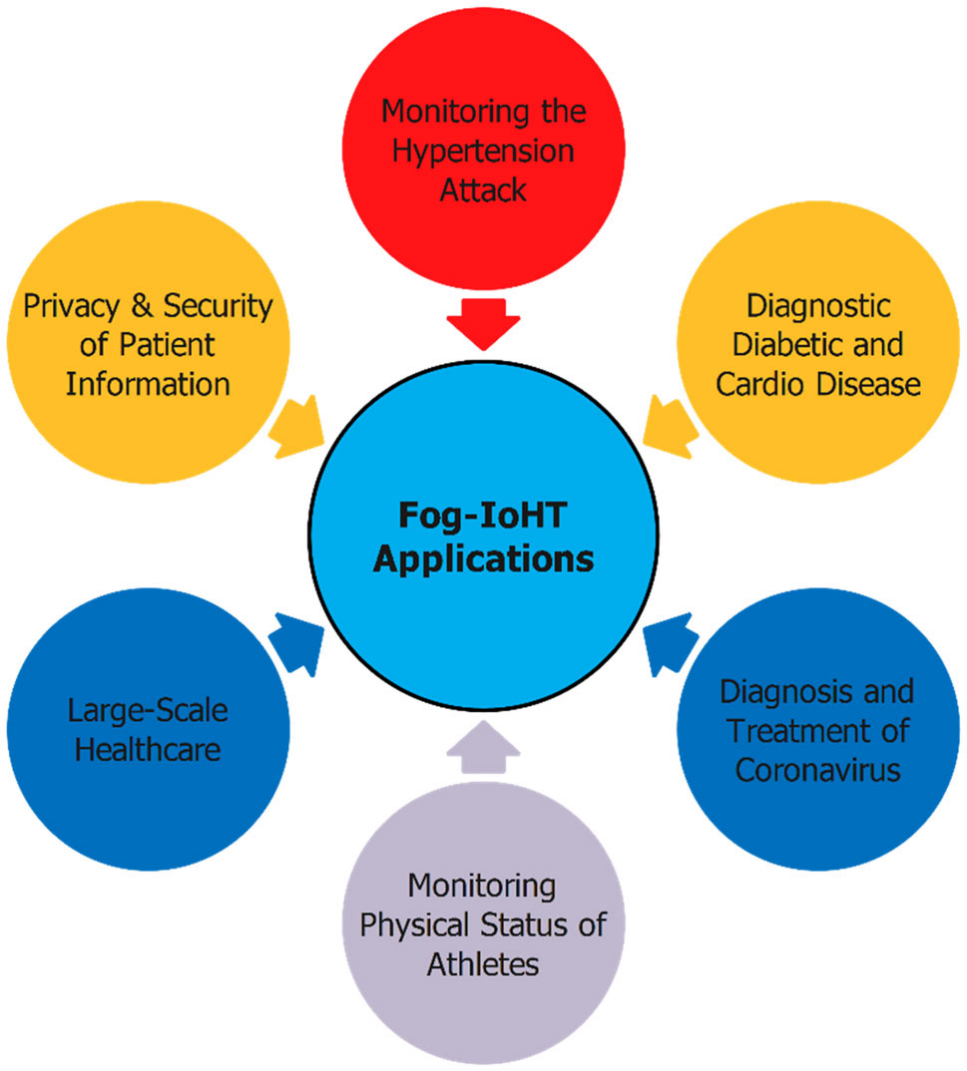
Several typical applications of fog-IoHT

Surveyed results indicated a series of the typical fog-IoHT applications as presented in Fig. 4, include monitoring hypertension attacks, diagnostic accuracy diabetic and cardio disease, diagnosis and treatment of infected patients coronavirus, etc. The survey results also indicated that many studies have been proposed for healthcare area within the past 3 years. One of the main purposes of fog computing is to move the abilities of the cloud closer to end-users for the purpose of the reduced service response times, enhance reliability, efficiency energy consumption, bandwidth, computing costs. The surveyed studies have achieved some positive results, however, there are still many challenges in taking fog computing into IoT applications [53, 54].

## Challenges and future research directions

**Fig. 5:**
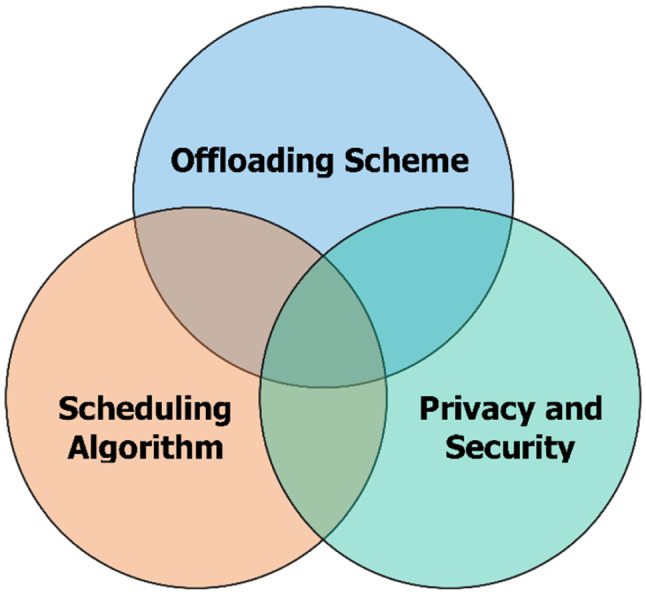
Some key challenges of IoHT applications

Nowadays, IoT is applied in almost all domains of humanity and it will play an extremely vital role as well as the key driving force for the development of the future Internet. In the next decade, billions of IoT devices and smart applications will emerge in a series of areas such as smart manufacturing, smart agriculture, augmented reality, healthcare, etc. and make basic changes for our world. To respond to these huge requirements, a robust, reliable, and flexible FC architecture to optimize network systems is topical and urgent. We discussed some challenges and possible research directions as presented in Fig. 5 for future edge computing as follows:

1. An efficient computing offloading scheme to achieve optimized performance needs to be considered. This scheme must be able to allocate appropriate tasks to both fog computing and cloud computing systems. In [55], the authors introduced a hybrid offloading schema between cloud and fog to tasks associated with complex applications. In this study, based on the task requirements, the IoT node based on the Q-learning algorithm will choose to offload tasks to the fog servers or to the cloud to load balancing and executed more efficiently.
2. An efficient scheduling algorithm to achieve energy efficiency, reliably manage and control fog servers in heterogeneous network environments needs to be considered. In [56], the authors introduced an efficient scheduling algorithm to saving energy for fog-based IoT systems. This algorithm aims to derive the optimal scheduling decision based on multiple neighbor helper nodes sharing their computation resources. Beside, in [57], the authors also introduced an efficient scheduling algorithm for efficient resource utilization on fog servers. Specifically, they rank fog nodes based on latency and resource utilization criteria. Then, intelligent scheduling algorithms based on the game theory approach are used to optimise the allocation of tasks.
3. In healthcare, the need for security on fog servers have been highlighted in recent studies due to potential unsafe issues from public network environments as well as resource sharing of servers [58, 59]. Accordingly to Turgut et al. [60] demonstrated that the successful deployment of the IoT applications will only happen if the customer benefits (the potential benefits that IoT systems provide to exceed their physical value and security and privacy costs). In our opinion, if privacy and security is not guaranteed, these potential risks can obscure the benefits of Internet of health Things applications [60]. Therefore, the privacy and security problem for fog-IoHT applications will continue to be the subject of attention.

The survey results have also indicated that integration between fog computing and IoHT applications is still in infancy stage; so a series of challenging problems need to be addressed in the future.

## Conclusions

The Internet of Things will be a comprehensive revolution of the future Internet. Based on IoT, the concepts of smart healthcare, smart cities, smart retail, smart agriculture and IoT systems are advent. To provide computing methods for these applications, several computational technologies have been proposed including cloud computing, edge computing, and fog computing. One of the most important applications of the IoT era is smart healthcare. A traditional healthcare application based on the cloud has some limitations such as high service response time and computing costs, more energy consumption and bandwidth. For the purpose of real-time ambulance emergency or rescue, rescue applications, these applications need real-time service response. Therefore, fog computing-based IoHT applications are proposed. The survey and analysis results have proven that fog computing-based IoHT applications reduce latency, save energy, improve performance, reliability compared to cloud computing. In reality, cloud computing is still a core computing technology. Fog computing was born to bring the capabilities of the cloud closer to end-users. Because the distance between the end-users and the database is shortened, fog computing has outstanding advantages over cloud computing and especially suitable for real-time IoHT applications. In this study, we propose an all-in-one computing architecture framework. Besides, we surveyed recently the IoT applications based on fog computing in the healthcare industry. Based on the survey results, we analyzed the application possibilities, challenges and future research directions. We hope the results of this study will be an important guide for future research in the field of fog computing and the healthcare industry.

## Data Availability

Not applicable.

## Appendix

See Table 3.

**Table 3:** Acronyms used in the survey and definitions

Acronym	Definition	Acronym	Definition
5G	5^th^ Generation Mobile Networks	IoMT	Internet of Medical Things
AI	Artificial Intelligence	IoT	Internet of Things
ANN	Artificial Neural Network	IoTH	Internet of Health Things
API	Application Programming Interface	IoV	Internet of Vehicles
AR	Augmented Reality	LAN	Local Area Network
CC	Cloud Computing	M2M	Mechanism to Mechanism
D2D	Device to Device	MANET	Mobile Ad hoc Networks
DDoS	Distributed Denial of Service	MCC	Multi-Cloud Computing
DoS	Denial of Service	MEC	Mobile Edge Computing
EG	Edge Computing	MILP	Mixed Integer Linear Programming
EMR	Electronic Medical Record	MOCSA	Multi-Objective Cuckoo Search Algorithm
FC	Fog Computing	MSSP	Multi-Stage Stochastic Programming
GIS	Geographic Information Systems	NDN	Named Data Networking
GPRS	General Packet Radio Service	PaaS	Platform as a Service
GPS	Global Positioning System	QoS	Quality of Service
IaaS	Infrastructure as a Service	SaaS	Software as a Service
IIoT	Industrial Internet of Things	SDN	Software-Defined Networking
IoHT	Internet of Health Things	UAV	Unmanned Aerial Vehicle
